# Twist and Polar Glide Symmetries: an Additional Degree of Freedom to Control the Propagation Characteristics of Periodic Structures

**DOI:** 10.1038/s41598-018-29565-6

**Published:** 2018-07-26

**Authors:** Fatemeh Ghasemifard, Martin Norgren, Oscar Quevedo-Teruel

**Affiliations:** KTH Royal Institute of Technology, Department of Electromagnetic Engineering, Stockholm, 10044 Sweden

## Abstract

New high-frequency 5G and satellite communication systems require fully-metallic antennas and electromagnetic components. These components can be implemented with truncated versions of periodic structures. In order to achieve the desired performance of these future devices, it is of crucial importance to have a precise control of the propagation properties, i.e. the frequency dispersion behavior and stop-bands. Here, we demonstrate the potential use of higher symmetries to diminish the frequency dispersion of periodic structures and control the width of stop-bands with a new type of fully-metallic transmission line, which is loaded with holes on a twist-symmetric configuration. Simulated and experimental results confirm the intrinsic link between the propagation characteristics and the symmetries of a periodic structure. Additionally, we provide a definitive explanation of the recently discovered polar glide symmetry and its potential combination with twist symmetries to produce low-dispersive materials and reconfigurable stop-bands. The promising properties of these structures are demonstrated with a fully-metallic reconfigurable filter, which could be used for future high-frequency 5G and satellite communication systems.

## Introduction

Periodic structures with higher symmetries are those that can be described by additional geometrical operations beyond the usual periodic condition^1^. Glide and twist (also named screw) symmetries are particular cases of higher symmetry^1–4^. A periodic structure with glide symmetry can be characterized by the geometrical operation G, which consists of half a period translation followed by a reflection with respect to a glide plane^2,3^. Moreover, a periodic structure with *m*-fold twist symmetry can be described by the geometrical operation S_*m*_, which consists of a *p*/*m* translation along and 2*π*/*m* rotation around the twist axis, where *p* is the periodicity of the structure and *m* is the degree of the twist symmetry^4^. One dimensional periodic structures with glide and twist symmetries were studied in the 1960’s and 70’s using the generalized Floquet theorem^1,5–7^. In these pioneering works, the symmetry properties of the structures were used to determine the characteristics of the guided and radiating fields. Recently, it has been demonstrated that applying higher symmetries to two dimensional periodic structures dramatically reduces their natural frequency dispersion^8,9^. Additionally, higher symmetries provide an additional degree of freedom to control the equivalent refractive index and stop-bands of periodic structures^4,10–12^. Due to these features, periodic structures with higher symmetries are an excellent candidate for producing ultra-wideband flat lenses^8,13,14^, low-dispersive leaky-wave antennas^15^, and low-loss high-frequency waveguide structures^16–19^.

Recently, the extraordinary properties of twist symmetries were empirically demonstrated with a transmission line loaded with twist-symmetric pins^4^. Moreover, in that work, polar glide symmetry was defined and combined with twist symmetry to produce a low dispersive periodic structure. However, that configuration did not allow a perfect implementation of polar glide symmetry^4^. Here, we demonstrate, with a loaded transmission line, the enormous potential of twist symmetries to produce low dispersive structures and to enable stop-bands in a given direction of propagation. We introduce, for the first time, a new kind of twist-symmetric structure that can be combined in an exact form with polar glide symmetry. Our results confirm the reduction in the frequency dispersion and also provide a definitive explanation of the effect of polar glide symmetry on the propagation characteristics. Our indications are corroborated with measurement results, and the potential of these structures is demonstrated with a filtering device. This filter is made of a fully-metallic structure that finds application in high frequency devices for example, in the future 5G communications^20^ or space technology^21^ (millimeter and sub-millimeter wave regimes), where the losses of dielectrics are prohibitive^9^.

## Results

### Twist symmetry effect

**Figure 1:**
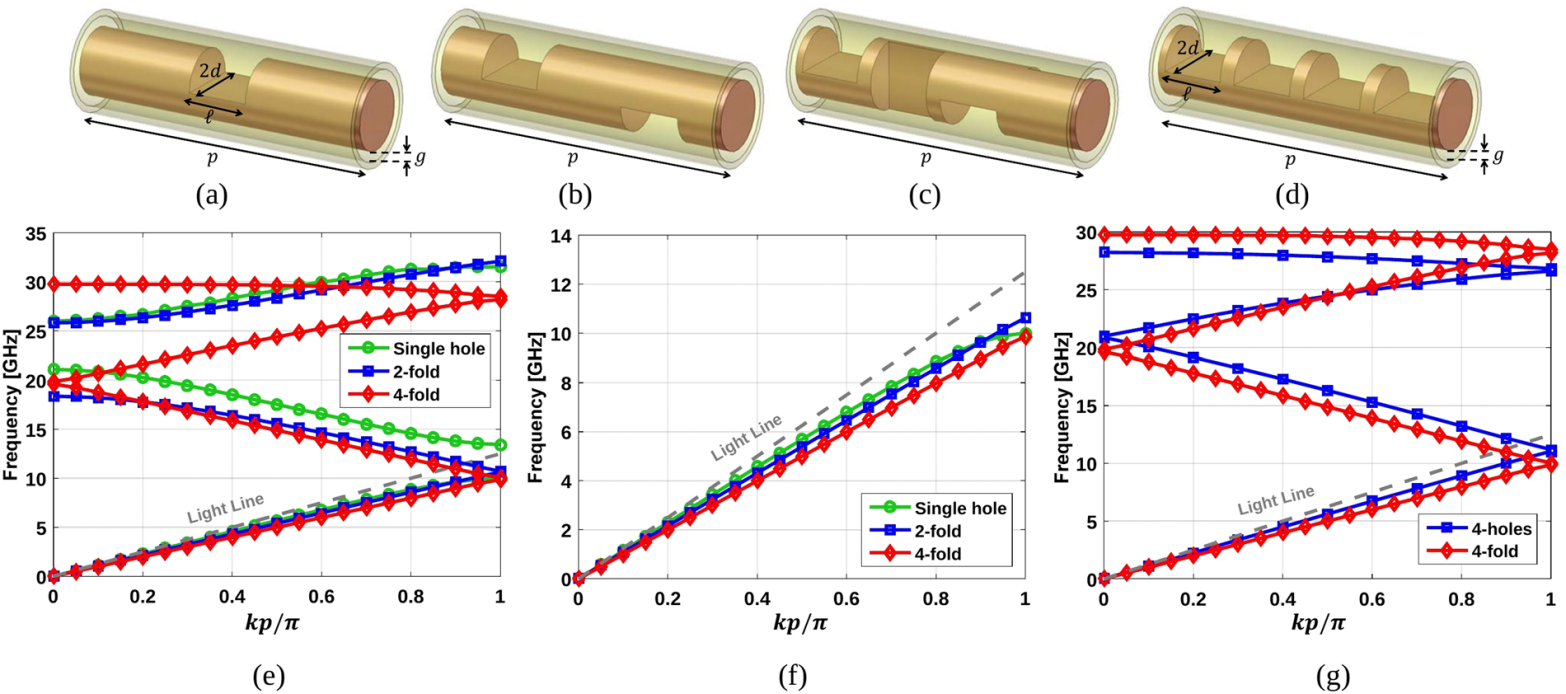
Unit cell of a coaxial line with (**a**) single hole, (**b**) 2-fold twist-symmetric, (**c**) 4-fold twist-symmetric, and (**d**) four holes. (**e**) Dispersion diagrams of the unit cells shown in (**a**–**c**); and (**f**) dispersion diagram of their first mode. (**g**) Dispersion diagrams of the unit cells shown in (**c**,**d**). The results in (**e**–**g**) correspond to the following parameters: *d* = 1.5 mm, $\ell =2.4$ mm, *p* = 12 mm, and *g* = 0.1 mm.

Let’s assume a coaxial transmission line that is composed of an inner conductor separated from an external conductor by a gap. One could add periodic holes on its inner conductor as shown in Fig. 1(a). Afterwards, one may add more holes in the inner conductor with a given translation and rotation, in order to create 2-fold and 4-fold twist-symmetric structures, as depicted in Fig. 1(b,c). Note that the holes are located in the middle of either the unit cell (Fig. 1(a)) or the sub-unit cells (Fig. 1(b,c)). To be able to compare the equivalent refractive indices of these cases, the same periodicity of the unit cell is assumed for all of them (*p* = 12 mm). The radii of inner and outer conductors are 1.5 mm and 1.6 mm. This means that the gap between the conductors denoted by *g* is 0.1 mm. A small gap is necessary to enforce a strong interaction between the two metallic surfaces that are confining the waves. The holes in all cases have the same size, where the length of the hole denoted by $\ell$ is 2.4 mm, and the opening angle of the holes is 180 degrees. This means that 2*d* in Fig. 1(a) is equal to the diameter of the inner conductor.

The simulated dispersion diagrams of these structures are illustrated in Fig. 1(e). For clarity, Fig. 1(f) shows only the first mode of the structures in a larger scale. When only one single hole is along the unit cell, a conventional plasmonic effect is found^22^. However, for the structures possessing twist symmetry, the frequency dispersion is almost completely removed for their first modes. The results in Fig. 1(f) also show that the equivalent refractive index increases when the degree of the twist symmetry increases too. In other words, by adding twist symmetry to a periodic structure and by increasing its degree, optically denser materials with lower frequency dispersion can be realised.

Considering the results in Fig. 1(e), shifting the frequency stop-band is possible by changing the degree of the twist symmetry of a periodic structure. Additionally, comparing the stop-band bandwidth for the cases with a single hole and 2-fold twist symmetry, a wider stop-band at higher frequencies is achieved in the latter. These phenomena can be explained with the generalized Floquet theorem^1,23^. This theorem states that the Bloch modes of a twist-symmetric structure are not only the eigenmodes of the translation operator T, but also the eigenmodes of the twist operator S_*m*_. This means that the fields in two adjacent sub-unit cells of a twist-symmetric structure are the same apart from an exponential factor exp(i*kp*/*m*)^23^. Therefore, the stop-band between the first and second modes of a conventional periodic structure will disappear by applying twist symmetry. This causes a wider stop-band at higher frequencies. By comparing the dispersion diagrams of the first three modes of the structures shown in Fig. 1(a,b), it can be seen that while the third mode is not affected considerably by the higher symmetry, the existence of the second hole in the 2-fold structure closes the first stop-band in the Brillouin zone, and shifts down the maximum propagating frequency of the second mode. Therefore, the total stop-band, which is established between the second and third modes, is wider in the structure with 2-fold twist-symmetric holes.

To complete the discussion on the twist symmetry effect, the dispersion diagrams of two unit cells of coaxial lines with four holes on their inner conductors without rotation (Fig. 1(d)) and with 90° rotation, which makes a 4-fold twist-symmetric structure (Fig. 1(c)) are obtained and compared in Fig. 1(g). The size of the holes, the gap, and the periodicity are the same in these two unit cells. Note that the real unit cell of the case shown in Fig. 1(d) is a coaxial line with the length of *p*/4 having a single hole on its inner conductor. However, for better comparison with the 4-fold twist-symmetric unit cell, four periods are considered, as illustrated in Fig. 1(d). The results depicted in Fig. 1(g) prove that with the same period and amount of material, a higher refractive index (in other words, an optically denser material) is achievable with a structure possessing higher symmetry compared to a conventional periodic structure.

**Figure 2:**
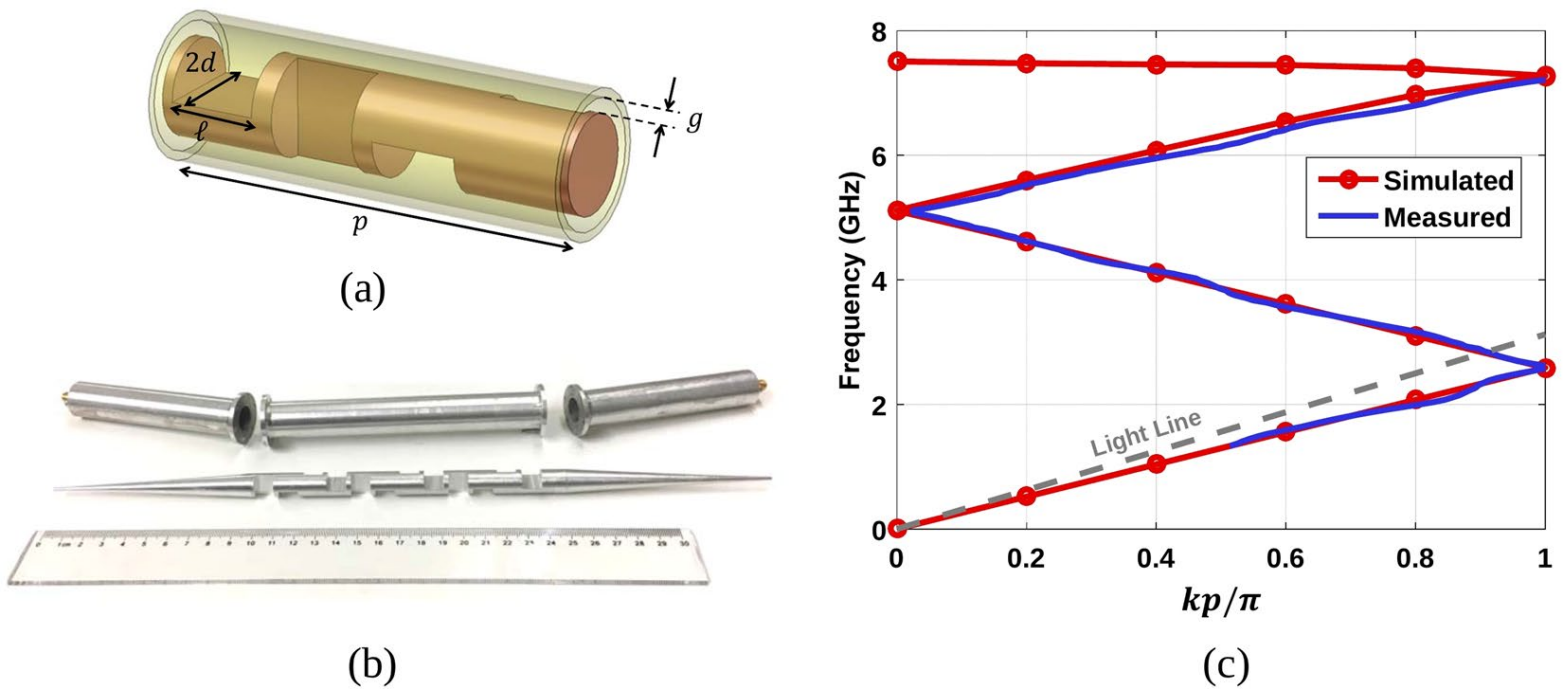
Realization of a coaxial transmission line with 4-fold twist-symmetric holes: (**a**) Twist-symmetric unit cell with dimensions, *d* = 6 mm, $\ell =9.6$ mm, *p* = 48 mm, and *g* = 1 mm. (**b**) Manufactured prototype consisting of three unit cells and the matching transitions. (**c**) Dispersion diagram of the unit cell obtained with the simulation and measurements.

To experimentally verify the simulation results, a prototype of a coaxial line loaded with 4-fold twist-symmetric holes has been manufactured and measured. The prototype is illustrated in Fig. 2(b) and consists of three unit cells with 4-fold twist symmetric holes, as the one shown in Fig. 2(a), and two transitions for impedance matching designed to operate at 1.5 GHz. The dimensions of the unit cell are: *d* = 6 mm, $\ell =9.6$ mm, *p* = 48 mm, and *g* = 1 mm. The scattering parameters of the whole structure and only the connected transitions were measured. To obtain the dispersion diagram of only the twist-symmetric unit cell, the effect of transitions were removed from the whole structure by post-processing. The comparison between the dispersion diagrams obtained by simulation and the measurement is depicted in Fig. 2(c). There is an excellent agreement for the frequency range from 1.5 GHz to 7 GHz.

### Polar glide symmetry

Polar glide is a particular case of glide symmetry in which the mirroring surface is a cylinder instead of a common flat surface (Cartesian glide)^4^. As mentioned, this idea was first introduced and applied to a coaxial transmission line loaded with pins^4^. However, in that work, a true mirroring with respect to a cylinder was not applied and a structure was called polar glide-symmetric if its flat approximation possessed Cartesian glide symmetry^4^. Here, we will investigate polar glide symmetry applied to a coaxial transmission line loaded with holes using mirroring with respect to a cylinder.

**Figure 3:**
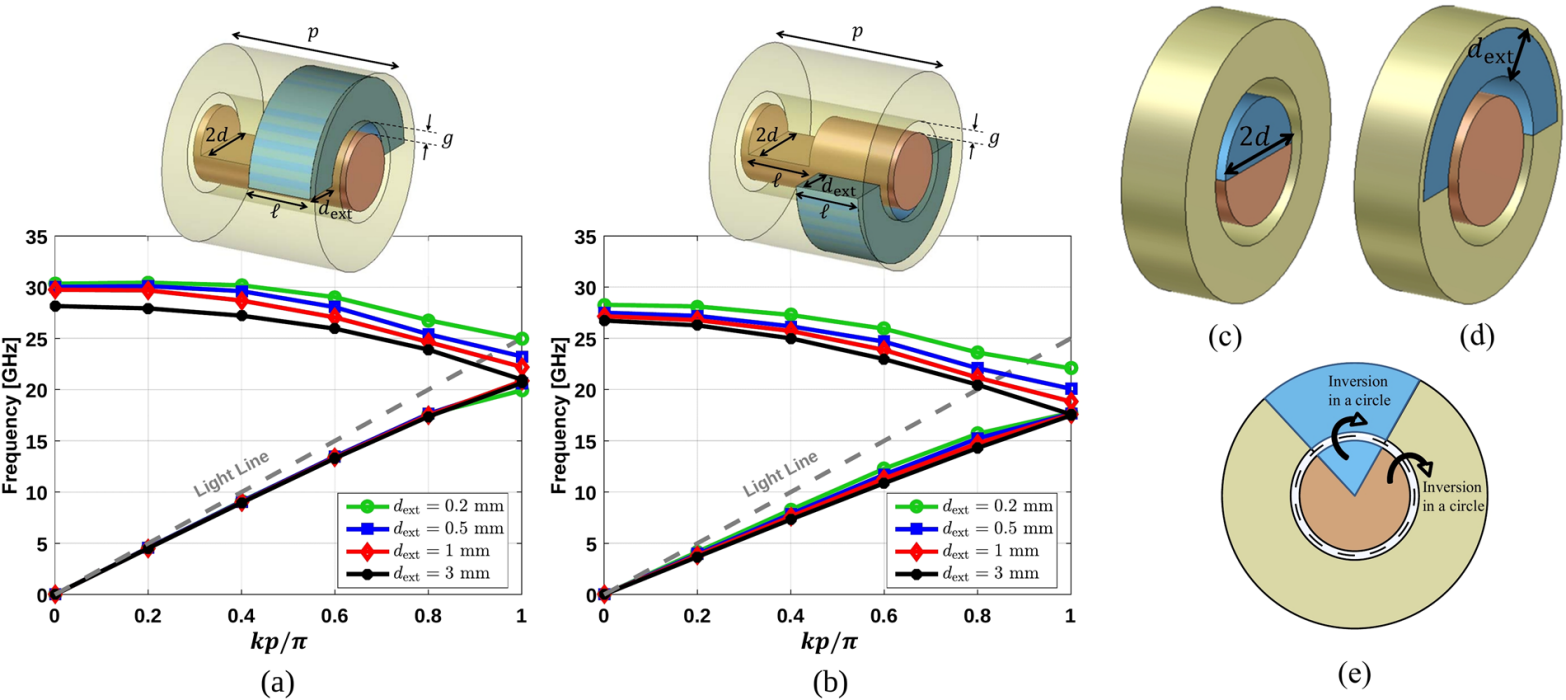
Unit cells and the dispersion diagrams of coaxial transmission lines with a hole in its inner conductor (shown transparent) and a hole in its outer conductor (shown in blue with a depth of *d*_ext_). The hole in the outer conductor has a *p*/2 translation and (**a**) 0° rotation (**b**) 180° rotation with respect to the hole in the inner conductor. The other parameters are *d* = 1.5 mm, $\ell =2.4$ mm, *p* = 6 mm, and *g* = 0.1 mm. (**c**) Cross section of a transmission line with a hole in its inner conductor and (**d**) its inversion in a circle, which has a hole with a depth of *d*_ext_ in its outer conductor. (**e**) Cross section of a conductor with a hole (in blue) and its inversion in a circle.

For this purpose, we consider two unit cells with a periodicity *p* as illustrated in Fig. 3(a,b). Each unit cell consists of two sub-unit cells with *p*/2 length. The first sub-unit cell has a hole in its inner conductor and the second one has a hole in its outer conductor. The length ($\ell$) and the opening angle (180°) of the holes are the same and they are located in the middle of the sub-unit cells. The radius of the inner conductor is *d* (Fig. 3(c)), the depth of the hole in the outer conductor is denoted by *d*_ext_ (Fig. 3(d)), and the gap between the conductors is denoted by *g*. Imagining a flat approximation of the cylinders, one could assume that the depth of the holes in the lower and upper planes should be equal to the radius of the inner conductor (*d*) and the depth of the hole in the outer conductor (*d*_ext_). Thus, since in the flat approximation the unit cell possesses Cartesian glide symmetry if *d* = *d*_ext_, it could be assumed that the original unit cell had polar glide symmetry if *d* = *d*_ext_. Also, in the work about the coaxial transmission line loaded with pins, the concept of polar glide was illustrated with pins of identical length protruding from the inner and outer conductors^4^. However, a glide operator must be defined as a combination of a translation (half of a periodicity) and a rotation followed by a reflection. In the case of polar glide, this reflection must be with respect to a cylindrical surface with a circular cross section, to ensure an equivalent response from inner and outer conductors. This cylindrical surface is located at the geometrical mean radius of the two coaxial conductors that is $\sqrt{d(d+g)}$ (see Fig. 3(e)). Therefore, the unit cells illustrated in Fig. 3(a,b) possess polar glide symmetry only when ${d}_{{\rm{ext}}}\to \infty$. A similar approach has been followed in transformation optics^24^.

The dispersion diagrams of the aforementioned unit cells for different values of *d*_ext_ are depicted in Fig. 3(a,b). The simulations were performed with the following parameters: *p* = 6 mm, $\ell =2.4$ mm, *d* = 1.5 mm, and *g* = 0.1 mm. These results reveal that polar glide symmetry is achieved when *d*_ext_ grows enough, and therefore, the stop-band at *k* = *π*/*p* is almost removed. Indeed, this is the same response as the one for periodic structures with Cartesian glide symmetry. Moreover, it is clear that by decreasing *d*_ext_, the polar glide symmetry is broken causing a stop-band between the first and second modes. This stop-band can be adjusted by changing *d*_ext_.

**Figure 4:**
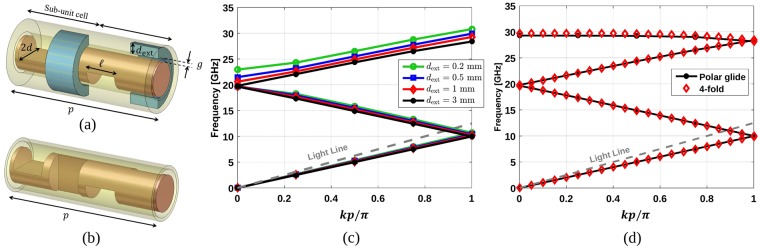
Unit cell of a coaxial transmission line with (**a**) 2-fold twist symmetry whose sub-unit cell has a hole in its inner conductor (shown transparent) and a hole in its outer conductor (shown in blue with a depth of *d*_ext_) and (**b**) 4-fold twist-symmetric holes. (**c**) Dispersion diagrams of the first structure for different values of *d*_ext_. The other parameters are *d* = 1.5 mm, $\ell =2.4$ mm, *p* = 12 mm, and *g* = 0.1 mm. (**d**) Comparison between the dispersion diagrams of these two structures when *d*_ext_ = 3 mm.

To demonstrate the possibility of designing reconfigurable filters by combining twist symmetry and polar-glide symmetry, a periodic structure with the unit cell shown in Fig. 4(a) is simulated. This unit cell possesses 2-fold twist symmetry, where its sub-unit cell has a hole in its inner conductor and a hole with a depth of *d*_ext_ in its outer conductor. This means that the sub-unit cell will have approximately polar glide symmetry if *d*_ext_ is large enough. In this case, polar glide symmetry is achieved with a *p*/4 translation, a 45° rotation and a reflection with respect to a cylindrical surface. The dispersion diagrams of this unit cell with *p* = 12 mm, $\ell =2.4$ mm, *d* = 1.5 mm, and *g* = 0.1 mm are shown in Fig. 4(c) for different values of *d*_ext_. As predicted, there is no stop-band between the first and second modes for any value of *d*_ext_ since the unit cell has 2-fold twist symmetry. However, there is a stop-band between the second and third modes, which is gradually removed when *d*_ext_ is large enough, i.e. the sub-unit cell has approached a perfect polar glide symmetry. Thus, we can conclude that the combination of polar glide symmetry and 2-fold twist symmetry mimics the behavior of 4-fold twist symmetry.

To further illustrate this phenomenon, the dispersion diagram of the unit cell shown in Fig. 4(a) with *d*_ext_ = 3 mm is compared with the case of the unit cell shown in Fig. 4(b), which is a 4-fold twist-symmetric periodic structure. The periodicity and the size of the holes are the same for these two unit cells. These results, illustrated in Fig. 4(d), confirm that the combination of polar glide and 2-fold twist symmetry presents a similar response as the 4-fold twist symmetry. At the end of this section, we demonstrate how this result can be used to design reconfigurable all-metal filters.

In summery, we can conclude that applying twist symmetry and/or polar-glide symmetry to a periodic structure provides a new degree of freedom to control its propagation characteristics, including the width and the location of the stop-bands. These properties open an opportunity for practical applications where fully metallic structures are needed for high-frequency antennas and electromagnetic components, such as phase shifters and filters. Here, we will illustrate how these structures can be used to produce a filter.

**Figure 5:**
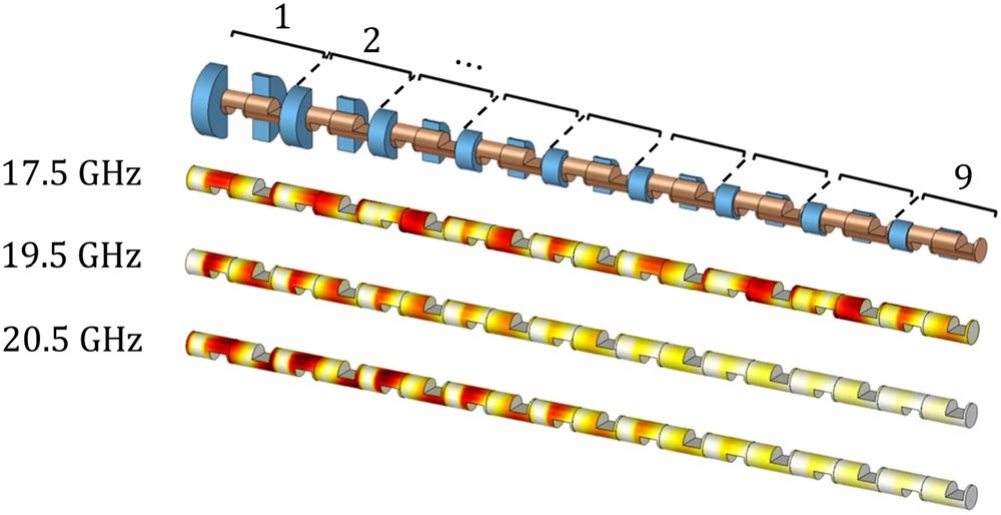
Simulated absolute value of the electric field of the wave propagating in the structure at three different frequencies. The structure consists of nine sections, where each section is a unit cell as shown in Fig. 4(a) with the exception that the depth of the holes in the outer conductor of two sub-unit cells is different and decreases gradually from 3 mm (section 1) to 0.2 mm (section 9). For representation purposes, the outer conductor has been hidden. The employed parameters are *d* = 1.5 mm, $\ell =2.4$ mm, *p* = 12 mm, and *g* = 0.1 mm as defined in Fig. 4(a).

As a first example, let us consider the structure depicted in Fig. 5. This structure consists of nine sections, where each section has the configuration of the unit cell already depicted in Fig. 4(a) with the parameters *d* = 1.5 mm, $\ell =2.4$ mm, *p* = 12 mm, and *g* = 0.1 mm, except that the depth of holes in the outer conductor of two sub-unit cells are different. In fact, *d*_ext_ decreases gradually from 3 mm to 0.2 mm. The rate of reduction is exponential for demonstration purposes. Note that in Fig. 5, the outer conductor is hidden for representation purposes. The electric field distributions of this structure at three frequencies (17.5, 19.5, and 20.5 GHz) are also shown in Fig. 5. At 17.5 GHz, the electromagnetic wave can propagate all along the structure since this frequency is located at the pass band of all sections (see Fig. 4(c)). However, at 19.5 and 20.5 GHz, the waves can only propagate a certain distance in the structure, being stopped in the unit cells in which there is a stop-band due to the lack of polar glide symmetry.

**Figure 6:**
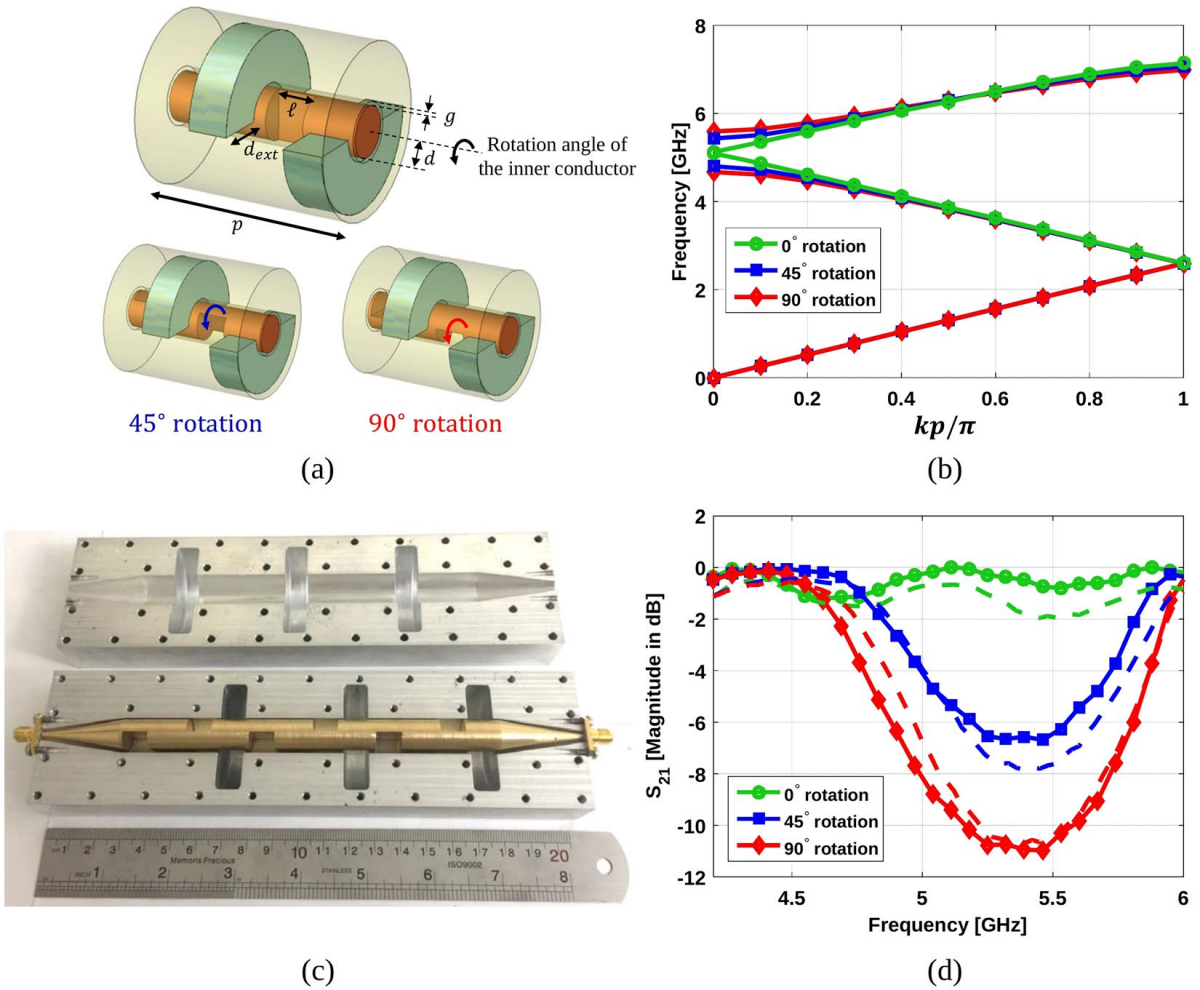
(**a**) Unit cell of a coaxial transmission line with 2-fold twist symmetry whose sub-unit cell possesses polar glide symmetry. The reference case (top) acts as a 4-fold twist-symmetric structure (0° rotation). In the bottom, two particular cases of broken 4-fold twist symmetry by rotating 45° and 90° of the inner conductor. (**b**) Simulated dispersion diagrams of the unit cells depicted in (a) when the parameters are *d* = 6 mm, *d*_ext_ = 12 mm, $\ell =9.6$ mm, *p* = 48 mm, and *g* = 1 mm. (**c**) Manufactured prototype consisting of three unit cells and the matching transitions. (**d**) Simulated (solid lines with markers) and measured (dashed lines) magnitude of the transmission coefficient S_21_ in dB.

Continuing with the idea of combining twist and polar glide symmetry, a reconfigurable fully-metallic filter has been designed and manufactured. The prototype is illustrated in Fig. 6(c) and consists of three unit cells and two matching transitions at the terminations. The unit cell possesses 2-fold twist symmetry and, additionally, the sub-unit cell has polar glide symmetry. Three different unit cell configurations are considered for our experiment as shown in Fig. 6(a): the reference case that behaves as a 4-fold twist-symmetric structure (0° rotation) and the cases with 45° and 90° rotation of the inner conductor. The parameters are chosen as *d* = 6 mm, *d*_ext_ = 12 mm, $\ell =9.6$ mm, *p* = 48 mm, and *g* = 1 mm. The dispersion diagram of these three unit cells, shown in Fig. 6(b), reveals that there is no stop-band between the second and third mode for the reference case. However, a stop-band is created and becomes wider by rotating the inner conductor. In other words, by rotating the inner conductor, the 4-fold twist symmetry of the reference case is broken, causing a stop-band between the second and third modes. This feature creates an opportunity for the design of fully-metallic reconfigurable filters.

The picture of the manufactured filter in Fig. 6(c) shows the filter in its reference situation (0° rotation). For demonstration purposes, the inner conductor can be rotated to produce the two other cases (45° and 90° rotation). The magnitude of the transmission parameter S_21_ for three different cases is illustrated in Fig. 6(d), including simulations and measurements. As predicted from the dispersion diagrams, at the frequencies between 4.75 GHz and 5.75 GHz, the electromagnetic waves are free to propagate for the 0° rotation, but they are stopped (filtered) for 45° and 90° rotation.

## Discussion

In this article, the effect of adding twist symmetries to periodic structures (here, a coaxial transmission line with periodic holes) has been investigated. We have demonstrated a link between symmetries (twist and polar glide) and the dispersive properties of a periodic structure. In particular, twist symmetry can be employed to reduce the frequency dispersion and change the location and the width of the stop-bands of a periodic structure. Additionally, by applying twist symmetry and by changing its degree, the equivalent refractive index of a periodic structure can be controlled. This effect can be employed to design, for example, fully-metallic leaky-wave antennas.

Furthermore, we have provided a final explanation of the recently discovered polar glide symmetry. We have demonstrated that by defining polar glide symmetry as a translation (with/without a rotation) followed by mirroring with respect to a cylindrical surface, an equivalent effect to Cartesian glide symmetry is obtained. The effect of applying both polar glide and twist symmetry to a periodic structure has been proposed and we have demonstrated that this combination of symmetries provides an additional degree of freedom to accurately control the propagation characteristics of periodic structures.

We have also provided measurement results of two prototypes to validate our conclusions, including a design of a practical device–a fully-metallic reconfigurable filter. The proposed fully-metallic filter combines both twist and polar glide symmetry and demonstrates that by breaking a higher symmetry, it is possible to create a stop-band in the dispersion diagram and, therefore, to stop the wave propagation in a desired band of frequencies. This filter benefits from being fully metallic which is an advantage in space applications and future 5G communications devices.

## Methods

The simulated dispersion diagrams were obtained using the Eigenmode Solver in *CST Microwave Studio*. The periodic boundary condition was selected along the coaxial line and perfect electric conductors were selected for two other directions, filling the gap between the outer conductors and the boundaries in *CST* with a perfect electric conductor. The Time Domain Solver and waveguide ports in *CST* were used to obtain the simulated transmission parameters needed for the results depicted in Figs 2(c) and 6(d). Moreover, the prototypes were manufactured from aluminum (Figs 2(b) and 6(c)) and brass (inner conductor of Fig. 6(c)) with a CNC machine. The measurement of transmission parameters was done using an Anritsu VNA (MS4640B Series). The post processing to exclude the effect of matching transitions for the results presented in Fig. 2(c) was performed in Matlab. For this purpose, two measurements were performed. First, the transmission coefficient of the whole structure shown in Fig. 2(b) was measured. Then, the transitions were connected to each other without the twist-symmetric unit cells and the transmission coefficient was measured. The difference between the phases of these two transmission coefficients represents the phase shift due to the main structure (the twist-symmetric coaxial line) that can be translated into the propagation constant of the structure under test.

### Data availability statement

The data generated and analysed during the study are available from the corresponding author on reasonable request.
